# Initial assessment of the infant with neonatal cholestasis—Is this biliary atresia?

**DOI:** 10.1371/journal.pone.0176275

**Published:** 2017-05-11

**Authors:** Benjamin L. Shneider, Jeff Moore, Nanda Kerkar, John C. Magee, Wen Ye, Saul J. Karpen, Binita M. Kamath, Jean P. Molleston, Jorge A. Bezerra, Karen F. Murray, Kathleen M. Loomes, Peter F. Whitington, Philip Rosenthal, Robert H. Squires, Stephen L. Guthery, Ronen Arnon, Kathleen B. Schwarz, Yumirle P. Turmelle, Averell H. Sherker, Ronald J. Sokol, for the Childhood Liver Disease Research Network

**Affiliations:** 1 Pediatric Gastroenterology, Hepatology, and Nutrition; Baylor College of Medicine; Houston, Texas, United States; 2 Department of Biostatistics; University of Michigan; Ann Arbor, Michigan, United States; 3 Children’s Hospital of Los Angeles; Los Angeles, California, United States; 4 Mount Sinai; New York, New York, United States; 5 University of Michigan Medical School; Ann Arbor, Michigan, United States; 6 Pediatric Gastroenterology, Hepatology, and Nutrition; Emory University School of Medicine/Children’s Healthcare of Atlanta; Atlanta, Georgia, United States; 7 Division of Gastroenterology, Hepatology, and Nutrition; Hospital for Sick Children and University of Toronto; Toronto, Ontario, Canada; 8 Pediatric Gastroenterology, Hepatology, and Nutrition; Indiana University School of Medicine/Riley Hospital for Children; Indianapolis, Indiana, United States; 9 Division of Pediatric Gastroenterology, Hepatology, and Nutrition; Cincinnati Children’s Hospital Medical Center; Cincinnati, Ohio, United States; 10 Division of Gastroenterology and Hepatology; University of Washington Medical Center; Seattle Children’s; Seattle, Washington, United States; 11 Pediatric Gastroenterology, Hepatology, and Nutrition; Children’s Hospital of Philadelphia; Philadelphia, Pennsylvania, United States; 12 Pediatrics Division of Gastroenterology, Hepatology, and Nutrition; Ann and Robert H. Lurie Children’s Hospital of Chicago; Chicago, Illinois, United States; 13 Division of Gastroenterology, Hepatology, and Nutrition; Department of Pediatrics; University of California San Francisco; San Francisco, California, United States; 14 Children’s Hospital of Pittsburgh; Pittsburgh, Pennsylvania, United States; 15 Pediatric Gastroenterology, Hepatology, and Nutrition; University of Utah; Salt Lake City, Utah, United States; 16 Johns Hopkins School of Medicine; Baltimore, Maryland, United States; 17 Washington University School of Medicine; St. Louis, Missouri, United States; 18 Liver Diseases Research Branch; National Institute of Diabetes and Digestive and Kidney Diseases; National Institutes of Health; Bethesda, Maryland, United States; 19 Section of Pediatric Gastroenterology, Hepatology, and Nutrition, Department of Pediatrics; University of Colorado School of Medicine; Children’s Hospital Colorado; Aurora, Colorado, United States; Texas A&M University, UNITED STATES

## Abstract

**Introduction:**

Optimizing outcome in biliary atresia (BA) requires timely diagnosis. Cholestasis is a presenting feature of BA, as well as other diagnoses (Non-BA). Identification of clinical features of neonatal cholestasis that would expedite decisions to pursue subsequent invasive testing to correctly diagnose or exclude BA would enhance outcomes. The analytical goal was to develop a predictive model for BA using data available at initial presentation.

**Methods:**

Infants at presentation with neonatal cholestasis (direct/conjugated bilirubin >2 mg/dl [34.2 μM]) were enrolled prior to surgical exploration in a prospective observational multi-centered study (PROBE–NCT00061828). Clinical features (physical findings, laboratory results, gallbladder sonography) at enrollment were analyzed. Initially, 19 features were selected as candidate predictors. Two approaches were used to build models for diagnosis prediction: a hierarchical classification and regression decision tree (CART) and a logistic regression model using a stepwise selection strategy.

**Results:**

In PROBE April 2004-February 2014, 401 infants met criteria for BA and 259 for Non-BA. Univariate analysis identified 13 features that were significantly different between BA and Non-BA. Using a CART predictive model of BA versus Non-BA (significant factors: gamma-glutamyl transpeptidase, acholic stools, weight), the receiver operating characteristic area under the curve (ROC AUC) was 0.83. Twelve percent of BA infants were misclassified as Non-BA; 17% of Non-BA infants were misclassified as BA. Stepwise logistic regression identified seven factors in a predictive model (ROC AUC 0.89). Using this model, a predicted probability of >0.8 (n = 357) yielded an 81% true positive rate for BA; <0.2 (n = 120) yielded an 11% false negative rate.

**Conclusion:**

Despite the relatively good accuracy of our optimized prediction models, the high precision required for differentiating BA from Non-BA was not achieved. Accurate identification of BA in infants with neonatal cholestasis requires further evaluation, and BA should not be excluded based only on presenting clinical features.

## Introduction

Neonatal cholestasis is a relatively common clinical issue that presents a complex diagnostic challenge for clinicians [1]. Cholestasis may not be readily identified at its onset and, as such, may present late in the course of the underlying disease process. An expansive differential diagnosis underlies the condition, which challenges one to prioritize diagnostic evaluations in order to sort through a complex set of etiologies in a relatively short time [2]. Shotgun approaches to diagnosis are typically not feasible in infants, while identification of life-threatening and treatable causes of cholestasis is a high priority. Newborn screening has the potential to identify some of the relevant disease processes.

One of the most important and relatively common specific causes of neonatal cholestasis is biliary atresia (BA). Timely diagnosis of BA is ultimately made by cholangiography at the time of exploratory laparotomy and histologic assessment of the surgically-removed bile duct remnant. Such timely diagnosis has the potential to improve clinical outcomes, as earlier hepatic portoenterostomy is associated with longer survival without liver transplantation [3]. Deciding which infants should undergo surgical exploration is critical. Ideally, one would like to minimize the number of infants who undergo unnecessary surgery, while not missing or delaying the diagnosis of BA. There is no universal consensus on the sequential steps to be taken in the diagnostic evaluation of neonatal cholestasis from the time of presentation leading up to exploratory surgery.

The Childhood Liver Disease Research Network (ChiLDReN), a National Institutes of Health-funded consortium, has conducted a prospective longitudinal study of 875 infants presenting with neonatal cholestasis at 15 clinical sites in the United States and Canada over an 11-year period. Data collected included details of the presenting clinical features, demographics, physical findings, laboratory values, and gallbladder sonography results that are typically available in routine clinical practice. Using these data, the objective of this study was to determine the predictive value for BA of typical testing performed in the evaluation of cholestatic infants prior to the decision for invasive testing (e.g., liver biopsy, cholangiography, exploratory laparotomy). A secondary goal was to develop a diagnostic algorithm to help guide the clinician’s decision-making for invasive testing.

## Materials and methods

### Study population

Between April 2004 and February 2014, infants presenting with neonatal cholestasis were enrolled in a prospective observational study of infants with cholestasis (PROBE: https://clinicaltrials.gov/ct2/show/NCT00061828, conducted by ChiLDReN). Written informed consent was obtained from the study participants’ parents or guardians, and the protocol was carried out under institutional review board (IRB) approval. Given the age of the participants, assent was not feasible. The IRB at each participating institution has approved PROBE (S1 Table). Inclusion criteria were: 1) age ≤180 days at presentation to a ChiLDReN center; and 2) serum direct or conjugated bilirubin >20% of total bilirubin (TB) and ≥2mg/dl. The PROBE protocol permitted the use of laboratory studies drawn prior to enrollment (“presentation”) to be used for inclusion criteria. Exclusion criteria were: 1) acute liver failure; 2) previous hepatobiliary surgery; 3) bacterial or fungal sepsis; 4) hypoxia, shock, or ischemic hepatopathy; 5) malignancy; 6) primary hemolytic disease; 7) drug or total parenteral nutrition-associated cholestasis; 8) extracorporeal membrane oxygenation (ECMO)-associated cholestasis; or 9) birth weight <1500g in an infant who did not have BA. Presenting clinical features (including stool color), demographics, physical findings, laboratory data, and gallbladder sonography findings were collected prospectively and recorded prior to the ultimate assignment of a clinical diagnosis. Evaluations of neonatal cholestasis were not prescribed and were according to local practice and conducted at local facilities.

Not all participants enrolled in PROBE were included in this analysis of predictors of BA. Participants were included only if they had laboratory studies indicating direct/conjugated hyperbilirubinemia that were performed at the time of “presentation” to the ChiLDReN clinical site. Inclusion in the BA cohort (Group 1) for this analysis required either the performance of a biliary drainage procedure for BA or exploratory surgery with the finding of an atretic extrahepatic bile duct by either inspection or attempted cholangiography. BA could not be definitively “confirmed” in infants who presented “late” in the clinical course and in whom clinicians determined that laparotomy or laparoscopy would not benefit the child or alter management. Inclusion in the Non-BA cohort (Group 2) required the identification of a specific alternative etiology for their cholestasis or cholangiography that excluded BA. For an infant with the clinical diagnosis of idiopathic neonatal hepatitis (INH) or idiopathic cholestasis (IC) to be included in this analysis, resolution of cholestasis was required as defined by a subsequent TB <1.0 mg/dL at >120 days of age (without hepatic portoenterostomy). INH was defined as neonatal cholestasis in which histologic evidence of giant cell hepatitis was present on liver biopsy and for whom no other etiology was confirmed. IC was defined as neonatal cholestasis that resolved in an infant who did not undergo liver biopsy or did not have giant cell hepatitis on a liver biopsy, and for whom no other etiology was confirmed. The outcome variable for this study is a confirmed study definition meeting diagnosis of BA or Non-BA (i.e., Group 1 vs. Group 2).

### Candidate predictors

Twenty-two variables collected at the time of the first evaluation at the ChiLDReN center were considered as candidate predictors, including age at disease onset and first evaluation, sex, race, ethnicity, anthropometrics (weight z-score, height z-score, head circumference z-score), palpable liver (including number of centimeters below the costal margin at the midclavicular line), palpable spleen, acholic stools, Alagille “syndromic” facial features, serum TB (defined as conjugated + unconjugated when total not measured), conjugated/direct bilirubin, alanine aminotransferase (ALT), aspartate aminotransferase (AST), alkaline phosphatase (ALP), gamma-glutamyl transpeptidase (GGTP), albumin, platelet count, cholesterol, and gallbladder sonography (presence or absence of the gallbladder, “small” gallbladder equated with presence). Age at first evaluation was defined as the earliest date among dates of study informed consent, diagnosis, or surgery; age at disease onset was defined as the earliest age at which there was caregiver reported icterus of eyes or skin, darkening of urine, or white/pale stools in the initial history case report form.

### Statistical analysis

Descriptive statistics for the characteristics listed above were provided for BA and Non-BA subjects included in the model development and those not included (Group 3 = BA not included and Group 4 = Non-BA not included). Differences between Groups 1 and 2 were assessed using two sample t-tests for the continuous parameters. Variables with skewed distributions were analyzed after first applying a log transformation, with the accompanying descriptive statistics reported on the original scale. Categorical variables were assessed using a Chi-Square test or Fisher’s exact test, where cell size(s) were ≤5 participants.

### Model development

Two types of model were used to find the best prediction models: a hierarchical classification and regression tree (CART) and a logistic regression model [4]. All 22 factors mentioned above were considered by both approaches, regardless of whether or not they obtained statistical significance in the univariate setting. CART analysis recursively partitions observations to define the optimum cutoff point for continuous predictors and identifies homogeneous groups having the largest difference in the outcome variable (minimum misclassification error rate). Each partition is a binary split based on a single independent variable. This process results in a classification rule with the optimum cut point for continuous variables and is represented as a tree. Once the full tree was grown, a pruning algorithm was run to avoid over-fitting. In the pruning process, the chi-square statistic for 2x2 contingency tables was calculated for each split. Using a pre-selected alpha level (p = 0.10), nodes whose chi-square values–as well as the chi-square values of subsequent splits–did not exceed the predetermined threshold were pruned.

A logistic regression prediction model was constructed using a forward stepwise hierarchical approach, with higher than standard p value, α = 0.10 [5–7]. To avoid losing study sample due to missing data, a sequential regression imputation method was used to impute missing values [8]. Only one randomly selected imputed data set was used for model development [9]. To define appropriate transformation of continuous variables, we used penalized-spline functions to explore the potential nonlinear effect of potential continuous predictors [10]. Potential interaction effects identified through CART analysis were considered in the model development process. The final model consists of only variables maintaining a 0.10 level of significance.

### Model evaluation

The ability of the multivariate model to correctly classify patients into the dichotomous disease classification (BA vs. Non-BA) was determined by assessing the area under the receiver operating characteristic (ROC) curve (AUC), where larger values on the 0–1 scale indicate greater concordance between the predicted and observed disease groups. Reapplying the model to our data, we further evaluated the disease misclassification rates at what are considered more definitive predicted probability thresholds.

The CART analysis was performed using R (version 3.2.2) software. Data imputation and all other analyses were conducted using SAS (version 9.3)[4].

## Results

During the study period, 875 infants with neonatal cholestasis were enrolled in PROBE. Strict criteria for BA and Non-BA inclusion were used in this analysis to increase the confidence for the predictive value of variables tested. Thus, 401 infants (Group 1) met criteria for the study definition of BA; 102 participants were classified clinically as BA by the study site, but after review of laboratory and operative data at presentation, these patients did not meet the strict study definition of BA and were excluded from analysis (Group 3: 58 excluded for lack of laboratory data at presentation and 44 for lack of operative demonstration of BA). Groups 1 and 3 were generally similar, except for a skewing of data to a “late” presentation in Group 3, which likely accounted for the decision to not proceed with hepatic portoenterostomy, thereby excluding those infants from Group 1 (S2 Table).

There were 259 of 372 infants enrolled in PROBE who did not have a clinical diagnosis of BA and met study criteria for Non-BA (Group 2). There were 113 infants (Group 4) with a clinical diagnosis of Non-BA excluded from analysis for potentially more than one reason, including: 1) inability to definitively exclude BA because, despite having a clinical diagnosis of indeterminate/IC, INH, choledochal cyst, or “other”, either TB was still elevated (>1 mg/dL) beyond 120 days of age and/or there was no cholangiographic evidence of bile duct patency; 2) laboratory data were not available at presentation; and 3) laboratory data at presentation did not meet PROBE entry criteria. Groups 2 and 4 were similar (S3 Table). The clinical phenotype in Group 4 may have been milder, with less apparent hepatomegaly and lower biochemical markers of liver disease (TB, direct bilirubin, conjugated bilirubin, ALT, and AST).

Diagnoses in the 259 Non-BA infants who met study criteria (Group 2) included IC (n = 72), INH (n = 61), alpha-1 antitrypsin deficiency (n = 31), Alagille syndrome (n = 28), panhypopituitarism (n = 12), cytomegalovirus infection (n = 10), bile duct paucity (n = 10), progressive familial intrahepatic cholestasis (n = 8), cystic fibrosis (n = 6), mitochondrial disease (n = 6), bile acid synthesis defect (n = 5), and other (n = 8; 1 each for hemophagocytic lymphohistiocytosis, hereditary spherocytosis, neonatal ascites, Caroli’s disease, perinatal sclerosing cholangitis, porphyria, hyperinsulinism, and duplicate gall bladder). The demographics, salient clinical features, and laboratory values of the BA and Non-BA groups obtained at presentation at the ChiLDReN sites are displayed in Table 1.

**Table 1:** Comparison of clinical information at presentation between infants with and without BA.

Variable	Group 1 (BA Included) % or Mean (SD) N = 401	Group (Non-BA Included) % or Mean (SD) N = 259	p-value
Race			0.130
White	244 (63%)	156 (61.2%)	
Black	61 (15.8%)	51 (20%)	
Asian	36 (9.3%)	13 (5.1%)	
Other	46 (11.9%)	35 (13.7%)	
Sex			**<0.001**
Male	191 (47.6%)	164 (63.3%)	
Female	210 (52.4%)	95 (36.7%)	
Ethnicity			0.146
Hispanic	92 (23%)	60 (23.3%)	
Non-Hispanic	308 (77%)	197 (76.7%)	
Age at First Evaluation (Days)	N = 401	N = 259	0.176
	63.5 (30.9)	60 (33.3)	
Age at Disease Onset (Days)	N = 401	N = 259	**<0.001**
	12.8 (18.5)	18.7 (22.1)	
Weight (kg)	N = 398	N = 257	**<0.001**
	4.5 (0.9)	4.1 (1.1)	
Length (cm)	N = 381	N = 252	**<0.001**
	55.5 (4)	54.3 (4.3)	
Head Circumference (cm)	N = 336	N = 215	**0.015**
	37.6 (2.2)	37.1 (2.6)	
Weight Z-Score	N = 398	N = 257	**<0.001**
	-1 (1)	-1.5 (1.2)	
Length Z-Score	N = 381	N = 252	**<0.001**
	-0.8 (1.5)	-1.4 (1.5)	
Head Circumference Z-Score	N = 336	N = 215	**0.026**
	-1.1 (1.6)	-1.4 (1.2)	
Acholic Stools			**<0.001**
Absent	69 (17.6%)	165 (66%)	
Present	322 (82.4%)	85 (34%)	
Acholic Stools (3 Levels)			**<0.001**
Normal	69 (17.6%)	165 (66%)	
White or Gray	184 (47.1%)	30 (12%)	
Pale	138 (35.3%)	55 (22%)	
Facial Features			**<0.001**
Normal	380 (95.2%)	207 (81.2%)	
Abnormal	19 (4.8%)	48 (18.8%)	
Liver Edge Palpable			**0.004**
Not Palpable	26 (7.3%)	34 (14.5%)	
Palpable	332 (92.7%)	201 (85.5%)	
Liver Edge Below Costal Margin (cm)	N = 334	N = 202	**<0.001**
	3.3 (1.6)	2.5 (1.4)	
Spleen Palpable			**0.018**
Not Palpable	188 (50%)	149 (59.6%)	
Palpable	188 (50%)	101 (40.4%)	
Direct Baseline Bilirubin (mg/dL)	N = 239	N = 162	0.335
	5.7 (2.2)	5.8 (3.2)	
Conjugated Baseline Bilirubin (mg/dL)	N = 215	N = 121	0.871
	4.3 (1.6)	4.6 (2.6)	
Total Baseline Bilirubin (mg/dL)	N = 401	N = 259	0.979
	8.3 (3.1)	8.6 (4.3)	
AST (u/L)	N = 397	N = 254	0.304
	232.1 (206.4)	284.2 (347.7)	
ALT (u/L)	N = 400	N = 255	0.230
	154.7 (124.3)	190.7 (232.5)	
Albumin (g/dL)	N = 391	N = 246	**0.006**
	3.6 (0.5)	3.5 (0.6)	
GGTP (u/L)	N = 379	N = 238	**<0.001**
	711.9 (537.5)	299 (380.5)	
Platelets (10^3^/ mm^3^)	N = 380	N = 243	**0.022**
	445.2 (180.2)	419.7 (197.3)	
Alkaline Phosphatase (IU/L)	N = 395	N = 254	0.230
	568.6 (320.7)	572.1 (252.1)	
Total Cholesterol (mg/dL)	N = 33	N = 54	0.925
	184.2 (61.3)	190.6 (82.3)	
Gallbladder			**<0.001**
Absent	125 (39.9%)	13 (6.5%)	
Present	5 (1.6%)	1 (1.4%)	
Present (Small)	142 (45.4%)	81 (40.7%)	
Normal	41 (13.1%)	105 (52.8%)	
Gallbladder (Absent vs. Present)			**<0.001**
Absent	125 (39.9%)	13 (6.5%)	
Present	188 (60.1%)	186 (93.5%)	

Univariate analysis identified 13 variables (Table 1), which were significantly different (**in bold**) between BA and Non-BA (Group 1 vs. Group 2), including age at disease onset, stool color, sex, facial features, weight z-score, length z-score, head circumference z-score, centimeters of liver palpable below the costal margin, palpable spleen, GGTP, albumin, platelet count, and gallbladder sonography. Infants with BA were more likely to have acholic stools, to be female, to be younger at disease onset, have greater z-score growth parameters, have normal facial features, more significant hepatosplenomegaly, a higher GGTP, albumin, and platelet count, and a sonographically absent gallbladder.

**Fig 1:**
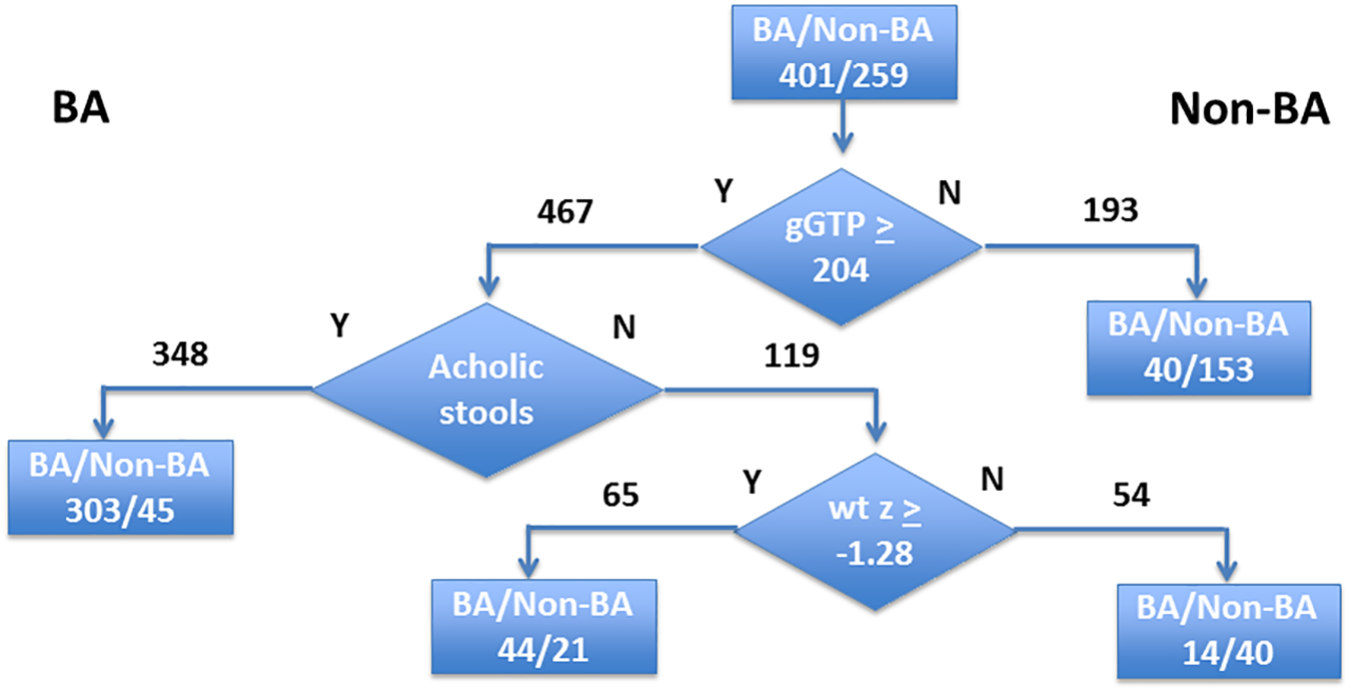
Hierarchical CART analysis of the prediction of BA. A pruned model is shown that uses GGTP level (cut-off 203.5 IU/L), acholic stools, and wt z-score (cut-off -1.28) to segregate BA from Non-BA as indicated.

We used a hierarchical CART analysis to create an algorithm that could distinguish BA from Non-BA. In this approach, the population was segregated into either BA or Non-BA in a stepwise manner based on the single most predictive variable, using a threshold value derived empirically from the observed data. After this initial segregation, each newly-created sub-population was again evaluated using the most predictive variable that was redefined for this new subset of the population. In this manner, the predictive power of each variable was maximized at each step. The process of segregation and reanalysis was continued until there was no further improvement in the overall predictive power for the population. The results of this analysis are shown in Fig 1.

If the initial discriminator was a GGTP of 204 IU/L, those with lower levels were unlikely to have BA (40 [21%] out of 193 infants). In those with GGTP ≥204 IU/L and acholic stools, BA was likely (303 out of 467 infants). Further discrimination was achieved by incorporating weight z-score. Overall, the predictive capacity for this model was somewhat worse than the logistic regression modeling, with an AUC for the ROC of 0.831. When the three-variable CART analysis was utilized, 12% of infants categorized as Non-BA (n = 247) were misclassified and had BA. Conversely, 17.5% of infants categorized as BA (n = 415) were misclassified and did not have BA.

The best logistic regression model selected included nine predictors: sex, acholic stools, normal facial features, ALT, GGTP, age at disease onset, weight z-score, palpable liver below the costal margin, and a sonographically absent gallbladder, which were associated with a diagnosis of BA (Table 2).

**Table 2:** Multivariate logistic regression analysis of factors predicting BA.

Variable	β	Odds Ratio	95% Confidence Interval	p-value
Intercept	-0.367			
Age at Onset (Days)	-0.011	0.989	(0.98, 1)	0.0514
Weight Z-Score	0.305	1.357	(1.11, 1.67)	0.003
Liver Below Costal Margin (cm)	0.320	1.377	(1.19, 1.60)	<0.0001
ALT (IU/L)	-0.002	0.998	(0.997, 1)	0.0265
GGTP(IU/L)	0.002	1.002	(1.001, 1.002)	<0.0001
Sex: Male vs. Female	-0.312	0.540	(0.35, 0.83)	0.0049
Acholic Stools:				<0.0001
Pale vs. Normal	0.252	4.775	(2.9, 7.88)	
White/Gray vs. Normal	1.061	10.725	(6.17, 18.66)	
Facial Features: Abnormal vs. Normal	-0.755	0.221	(0.10, 0.48)	0.0001
Gallbladder: Present vs. Absent	-0.820	0.194	(0.11, 0.35)	<0.0001

**Fig 2:**
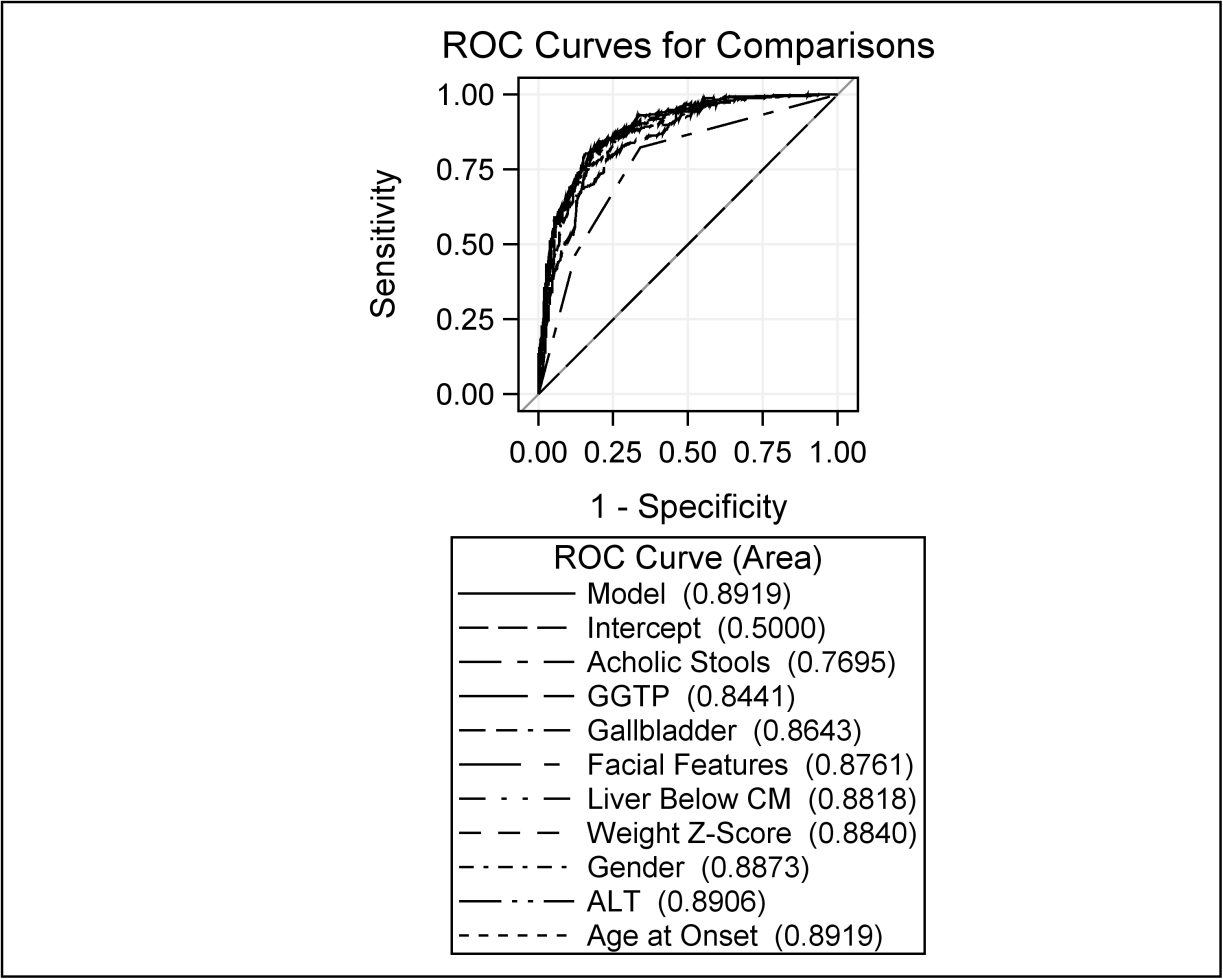
Receiver operator curve analysis of a multivariate model to predict the diagnosis of BA. The blue solid line is for the final nine-level model. The rest of the curves indicate AUC for a series of models obtained in the stepwise selection procedure. In stepwise order: intercept only, acholic stools, GGTP, gallbladder absence, absence of abnormal facial features, centimeters of liver palpable below the costal margin, weight z-score, sex, ALT, and age of disease onset.

Model discriminating ability was assessed by the ROC curve. Larger values on the 0–1 scale indicated a better predictive model. The final model yielded an AUC for the ROC analysis of 0.892 (Fig 2).

**Fig 3:**
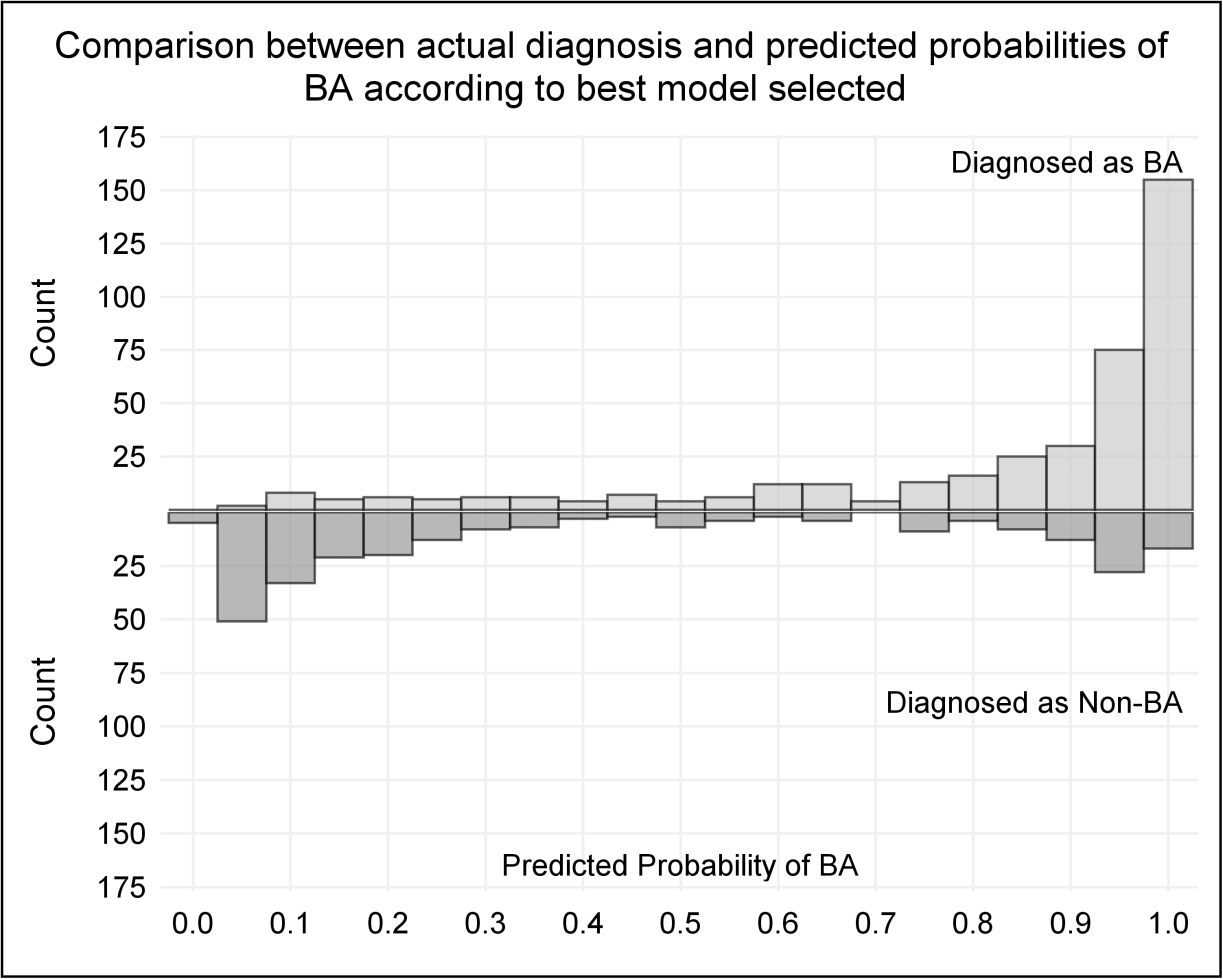
Logistic regression model of predicted probability of BA. Based upon a nine-feature model, a predicted probability of BA was calculated for each participant, with increased probability of BA as the score increased from 0 to 1. The number of participants with the probability scores is shown on the figure, with those with BA above the horizontal line and those with Non-BA below the line.

If all 22 candidate predictor features were incorporated into the model, the AUC of the ROC increased marginally to 0.898. Based upon the final model [logit(p) = -0.367–0.011*Age at Onset (Days) + 0.305*Weight Z-Score + 0.320*Liver Below Costal Margin—0.002*ALT(IU/L) + 0.002*GGTP (IU/L)—0.312*Male + 0.252*Pale Stools + 1.061*White/Gray Stools—0.755*Abnormal Facial Features—0.820*Present Gallbladder], a predicted probability of BA was calculated, with 1 indicating the highest chance (100%) of being BA, and 0 being the lowest (0%). The distribution of predicted probabilities for BA and actual study diagnoses of BA and Non-BA are displayed in Fig 3.

Three-hundred fifty-seven infants had a predicted probability >0.8, of whom 290 had BA (81.2%). Of the 67 remaining Non-BA infants (19%) with a predicted probability of >0.8, 12 had alpha-1 antitrypsin deficiency, and 10 had Alagille syndrome (Table 3). One-hundred thirty-six infants had a predicted probability of <0.2, of whom 120 had Non-BA (88.2%). Sixteen infants (12%) with scores <0.2 had BA and were evaluated at mean of 63 days of age; most had normally pigmented stools and gallbladder that was present. One-hundred sixty-seven infants had intermediate predicted probability scores between 0.2 and 0.8.

**Table 3:** Demographics, clinical, and laboratory profile of infants with BA predicted probability >0.8 or <0.2 (BA vs. Non-BA).

Predicted Probability	Parameter	Group 1 (BA)		Group 2 (Non-BA)		Overall	
		N	Mean (SD) or %	N	Mean (SD) or %	N	Mean (SD) or %
≥0.80	Age at First Evaluation (Days)	290	64.6 (31.2)	67	60.6 (36.6)	357	63.9 (32.3)
	Age at Disease Onset (Days)		11.8 (17.2)		15.7 (22.1)		12.5 (18.3)
	Weight Z-Scores		-0.8 (1.0)		-1.3 (1.0)		-0.9 (1.0)
	Liver Edge Below Costal Margin (cm)		3.4 (1.7)		2.6 (1.5)		3.2 (1.7)
	ALT		155 (111)		191 (169)		162 (124)
	GGTP		810 (566)		478 (543)		748 (576)
	Sex						
	Male	126	43.5%	39	58.2%	165	46.2%
	Female	164	56.6%	28	41.8%	192	53.8%
	Acholic Stools						
	Normal	9	3.1%	4	6.0%	13	3.6%
	White or Gray	137	47.2%	7	10.5%	144	40.3%
	Pale	144	49.7%	56	83.6%	200	56.0%
	Facial Features						
	Normal	276	95.2%	54	80.6%	330	92.4%
	Dysmorphic	14	4.8%	13	19.4%	27	7.6%
	Gallbladder						
	Absent	142	49.0%	11	16.4%	153	42.9%
	Present	148	51.0%	56	83.6%	204	57.1%
	Diagnosis						
	BA	285	98.3%	0	0	285	79.8%
	Alpha1-Antitrypsin Deficiency	0	0	12	17.9%	12	3.4%
	Hereditary Fructose Intolerance	0	0	1	1.5%	1	0.3%
	Cystic Fibrosis	0	0	1	1.5%	1	0.3%
	PFIC 1, 2, or 3	0	0	1	1.5%	1	0.3%
	Alagille Syndrome	1	0.3%	10	14.9%	11	3.1%
	Operable-Extrahepatic BA and Choledochal Cyst	2	0.7%	0	0	2	0.6%
	INH	0	0	15	22.4%	15	4.2%
	Cholestasis, Indeterminate	0	0	18	26.9%	18	5.0%
	Other	1	0.3%	8	11.9%	9	2.5%
	Choledochal Cyst	1	0.3%	1	1.5%	2	1.5%
≤0.20	Age at First Evaluation (Days)	16	62.8 (30.8)	120	56.7 (30.3)	136	57.4 (30.3)
	Age at Disease Onset (Days)		29.0 (31.4)		22.7 (23.2)		23.4 (24.3)
	Weight Z-Scores		-1.8 (1.1)		-1.8 (1.1)		-1.8 (1.1)
	Liver Edge Below Costal Margin (cm)		1.9 (1.5)		1.9 (1.1)		1.9 (1.1)
	ALT		199 (202)		194 (285)		194 (276)
	GGTP		289 (174)		182 (183)		194 (185)
	Sex						
	Male	6	37.5%	84	70.0%	90	66.2%
	Female	10	62.5%	36	30.0%	46	33.8%
	Acholic Stools						
	Normal	15	93.8%	119	99.2%	134	98.5%
	White or Gray	1	6.3%	1	0.8%	2	1.5%
	Pale	0	0	0	0	0	0
	Facial Features						
	Normal	15	93.8%	95	79.2%	110	80.9%
	Dysmorphic	1	6.3%	25	20.8%	26	19.1%
	Gallbladder						
	Absent	1	6.3%	2	1.7%	3	2.2%
	Present	15	93.8%	118	98.3%	133	97.8%
	Diagnosis						
	BA	12	75.0%	0	0	12	8.8%
	Alpha1-Antitrypsin Deficiency	0	0	9	7.5%	9	6.6%
	Storage Diseases	1	6.3%	0	0	1	0.7%
	Cystic Fibrosis	0	0	1	0.8%	1	0.7%
	PFIC 1, 2, or 3	0	0	2	1.7%	2	1.5%
	Alagille Syndrome	1	6.3%	5	4.2%	6	4.4%
	Bile Acid Synthetic Disorder	0	0	1	0.8%	1	0.7%
	INH	0	0	32	26.7%	32	23.5%
	Cholestasis, Indeterminate	0	0	36	30.0%	36	26.5%
	Other	0	0	28	23.3%	28	20.6%
	Hepatitis Due to CMV	0	0	6	5.0%	6	4.4%
	Choledochal Cyst	2	12.5%	0	0	2	1.5%

## Discussion

The quest for finding clinical and laboratory features that distinguish BA from other causes of neonatal cholestasis has been ongoing for over 50 years [11–20]. Early investigations of over 800 infants in five separate reports from Boston, Toronto, London, Houston, and Bicêtre demonstrated a difficulty in clinically distinguishing BA from intrahepatic cholestasis in a significant number of infants [11–15]. Infants with BA more frequently had acholic stools, had less failure to thrive, and had more pronounced elevation in biochemical markers of bile duct and canalicular injury, although these features were not uniformly discriminative. More recent reports have added radiologic and histologic features to the investigative paradigm [17–19]. Most of these studies have been single or two-center studies and retrospective in nature.

The current analysis is based on data obtained in a large, truly multi-centered prospective study, which was particularly rigorous with regard to the study definition of BA and Non-BA and with the application of advanced statistical modeling methods. The purpose of the current study was to attempt to develop a diagnostic algorithm that could distinguish between BA and Non-BA using non-invasive parameters that were typically obtained during initial clinical evaluation of cholestatic infants. An effective algorithm might serve as a guide to physicians as to whether invasive procedures, such as liver biopsy and exploratory laparotomy, are warranted. The three variables in the CART analysis (serum GGTP, acholic stools, and weight z-score) that were statistically derived to achieve the best prediction of BA are simple, mostly objective, and readily available early in the course of the evaluation of cholestasis. Accurate classification of the stool pigmentation is the only somewhat subjective parameter in this algorithm [21]; however, recent simple smartphone technology may overcome this [22]. The predicted probability model that was developed achieved accurate diagnosis of BA in 290 out of 357 cases (81%) when the predictive probability was >0.8. Accuracy in these cases might be enhanced if alpha-1 antitrypsin levels and phenotype were readily available, and if features of Alagille syndrome were carefully assessed. One could argue that, for the infants with a predictive probability of >0.8 who had negative diagnostic testing for alpha-1 antitrypsin deficiency and Alagille syndrome, the next logical step would be exploratory laparotomy, and one might defer liver biopsy. An accurate diagnosis of Non-BA was predicted in 120 of 136 cases (88%) when the predicted probability was <0.2. Conversely, an unsettling number of these infants had BA, whose diagnosis would be delayed or missed if one relied solely on these presenting clinical features to “exclude” BA. In addition, a significant number of infants had intermediate predicted probability scores between 0.2 and 0.8 and could not be classified as either BA or Non-BA.

It is clear from the current detailed analysis that clinicians should be very cautious about either diagnosing or excluding BA on the basis of presenting clinical features in infants with cholestasis. Family history is typically noninformative, but in selected circumstances can direct investigations toward specific inherited disorders like Alagille syndrome or familial intrahepatic cholestasis. Additional diagnostic investigations are typically warranted, and noninvasive approaches are often the first to be considered [23]. In the current study, only the presence of gallbladder was considered on ultrasonography. More detailed evaluation for the triangular cord sign, gallbladder wall characteristics, and hepatic subcapsular blood flow were not conducted, although may have increased the accuracy of the predictive model [18, 24–26]. Hepatobiliary scintigraphy may be especially useful in excluding BA when intestinal excretion of radiotracer is demonstrated, although nonexcretion is less helpful since it is observed in BA and Non-BA [27]. Thus, in 60 of 67 cases where a predictive value of >0.8 erroneously suggested BA, stools were pale or normal; in such infants, hepatobiliary scintigraphy may have been useful.

The current analysis did not attempt to determine the added value of liver histology in the predictive algorithm, as the focus was to determine the predictive value of tests performed prior to subjecting infants to invasive testing. Liver histology can be quite informative in the evaluation of neonatal cholestasis, although false negative rates are disturbing given the consequences of late or missed diagnosis of BA [28, 29]. In addition, the exposure of infants unnecessarily to anesthesia (for liver biopsy, cholangiography, or laparotomy) has become a relevant issue in light of recent reports of potential long-term neurodevelopmental sequelae of general anesthesia in young children [30]. Clinicians should consider this issue when deciding about diagnostic testing that may require general anesthesia, including liver biopsy and endoscopic, percutaneous, or intraoperative cholangiography.

## Conclusions

In conclusion, early accurate diagnosis of BA remains challenging. Clinicians are obliged to categorically exclude BA in the setting of neonatal cholestasis, since failure to make this diagnosis has potentially profound adverse consequences. This rigorous prospective analysis of presenting features in neonatal cholestasis was unable to generate a diagnostic algorithm that yielded sufficient ability to discriminate between BA and Non-BA in all patients. Early referral to a specialist, with consideration for possible liver biopsy or intraoperative cholangiography, needs to be entertained as soon as cholestasis is identified. Caution should be exercised in excluding BA based only on clinical non-invasive features. The identification of an alternative definitive diagnosis makes BA unlikely, although the Kasai hepatoportoenterostomy has been performed mistakenly in some infants with alternative diagnoses, including cystic fibrosis, alpha-1 antitrypsin deficiency, and Alagille syndrome [31–35]. Although not necessary for all infants with neonatal cholestasis, surgical exploration with operative cholangiography and/or pathologic examination of a bile duct remnant remains the only definitive means of making the diagnosis of BA.

## Supporting information

S1 Table Institutional review boards. (DOCX)

S2 Table Comparison of included (Group 1) and excluded (Group 3) infants with a clinical diagnosis of biliary atresia. (DOCX)

S3 Table Comparison of included (Group 2) and excluded (Group 4) infants with a clinical diagnosis that was not biliary atresia. (DOCX)
