# Supplementary material for: Initial assessment of the infant with neonatal cholestasis—Is this biliary atresia?
Source: PLoS One. 2017 May 11;12(5):e0176275. doi: 10.1371/journal.pone.0176275 (PMC5426590; doi:10.1371/journal.pone.0176275)
Supplement: S1 Table — (DOCX) [file pone.0176275.s001.docx]

Supporting Information for:

**Initial assessment of the infant with neonatal cholestasis – is this biliary atresia?**

Benjamin L. Shneider^1*^, Jeff Moore^2^, Nanda Kerkar^3,4^, John C. Magee^5^, Wen Ye^2^, Saul J. Karpen^6^, Binita M. Kamath^7^, Jean P. Molleston^8^, Jorge A. Bezerra^9^, Karen F. Murray^10^, Kathleen M. Loomes^11^, Peter F. Whitington^12^, Philip Rosenthal^13^, Robert H. Squires^14^, Stephen L. Guthery^15^, Ronen Arnon^4^, Kathleen B. Schwarz^16^, Yumirle P. Turmelle^17^, Averell H. Sherker^18^, Ronald J. Sokol^19^ for the Childhood Liver Disease Research Network.

^1^ Pediatric Gastroenterology, Hepatology, and Nutrition; Baylor College of Medicine; Houston, Texas, United States

^2^ Department of Biostatistics; University of Michigan; Ann Arbor, Michigan, United States

^3^ Children’s Hospital of Los Angeles; Los Angeles, California, United States

^4^ Mount Sinai; New York, New York, United States

^5^ University of Michigan Medical School; Ann Arbor, Michigan, United States

^6^ Pediatric Gastroenterology, Hepatology, and Nutrition; Emory University School of Medicine/Children’s Healthcare of Atlanta; Atlanta, Georgia, United States

^7^ Division of Gastroenterology, Hepatology, and Nutrition; Hospital for Sick Children and University of Toronto; Toronto, Ontario, Canada

^8^ Pediatric Gastroenterology, Hepatology, and Nutrition; Indiana University School of Medicine/Riley Hospital for Children; Indianapolis, Indiana, United States

^9^ Division of Pediatric Gastroenterology, Hepatology, and Nutrition; Cincinnati Children’s Hospital Medical Center; Cincinnati, Ohio, United States

^10^ Division of Gastroenterology and Hepatology; University of Washington Medical Center, Seattle Children’s, Seattle, Washington, United States

^11^ Pediatric Gastroenterology, Hepatology, and Nutrition; Children’s Hospital of Philadelphia; Philadelphia, Pennsylvania, United States

^12^ Pediatrics Division of Gastroenterology, Hepatology, and Nutrtion; Ann and Robert H. Lurie Children’s Hospital of Chicago; Chicago, Illinois, United States

^13^ Division of Gastroenterology, Hepatology, and Nutrition; Department of Pediatrics; University of California, San Francisco; San Francisco, California, United States

^14^ Children’s Hospital of Pittsburgh; Pittsburgh, Pennsylvania, United States

^15^ Pediatric Gastroenterology, Hepatology, and Nutrition; University of Utah, Salt Lake City, Utah, United States

^16^ Johns Hopkins School of Medicine; Baltimore, Maryland, United States

^17^ Washington University School of Medicine, St. Louis, Missouri, United States

^18^ Liver Diseases Research Branch, National Institute of Diabetes and Digestive and Kidney Diseases, National Institutes of Health; Bethesda, Maryland, United States

^19^ Section of Pediatric Gastroenterology, Hepatology, and Nutrition, Department of Pediatrics, University of Colorado School of Medicine; Children’s Hospital Colorado; Aurora, Colorado, United States

***Corresponding Author:** Benjamin L. Shneider: [Benjamin.Shneider@bcm.edu](mailto:Benjamin.Shneider@bcm.edu)

**S1 Table. Institutional Review Boards**

| **Emory University** Institutional Review Board 1599 Clifton Road, 5th Floor Atlanta, Georgia 30322 Tel: 404-712-0720 Fax: 404-727-1358 Email: [irb@emory.edu](mailto:irb@emory.edu) Web: <http://www.irb.emory.edu/> |
| --- |
| **Johns Hopkins Medicine** Office of Human Subjects Research Institutional Review Boards 1620 McElderry Street, Reed Hall, Suite B-130 Baltimore, Maryland 21205-1911 Tel: 410-955-3008 Fax: 410-955-4367 Fax Email: [jhmirb@jhmi.edu](mailto:jhmirb@jhmi.edu) |
| **Cincinnati Children’s** Office of Research Compliance & Regulatory Affairs 3333 Burnet Avenue Suite R3325 MLC 7040 Cincinnati, OH 45229-3039 Tel: 513-636-2754 Fax: 513-636-1321 Email: [orcra@cchmc.org](mailto:orcra@cchmc.org) |
| Colorado Multiple Institutional Review Board, CB F490 **University of Colorado**, Anschutz Medical Campus 13001 E. 17th Place, Building 500, Room N3214 Aurora, Colorado 80045 Tel: 303-724-1055 Fax: 303-724-0990 Email: [comirb@ucdenver.edu](mailto:comirb@ucdenver.edu) Web: uchsc.edu/comirb Federalwide Assurance (FWA): FWA00005070 |
| **Baylor College of Medicine** Office of Research One Baylor Plaza, 600D Houston, Texas 77030 Tel: 713-798-6970 Fax: 713-798-6990 Email: [irb@bcm.tmc.edu](mailto:irb@bcm.tmc.edu) |
| **Indiana University** Office of the Vice President for Research Office of Research Compliance 980 Indiana Ave., LV 3315 Indianapolis, IN 46202 Tel: 317-274-8289 (Indianapolis) or 812-856-4242 (Bloomington) Email: [irb@iu.edu](mailto:irb@iu.edu) |
| **Children’s Hospital Los Angeles** Committee on Clinical Investigations (CCI) Institutional Review Board (IRB) CHLA Institutional Review Board Human Subjects Protection Program 4650 Sunset Boulevard, Mailstop #23 Los Angeles, CA 90027-6062 Tel: 323-361-2265 Fax: 323-361-3620 Web: <http://www.childrenshospitalla.org/body.cfm?id=213> FWA: 00001914 |
| Program for the Protection of Human Subjects **Mount Sinai School of Medicine and Mount Sinai Hospital** One Gustave L. Levy Place, Box 1075 3 East 101^st^ Street, First Floor New York, NY 10029-6530 Tel: 212-824-8200 Fax: 212-876-6786 |
| **The Children’s Hospital of Philadelphia** Research Institute The Committee for the Protection of Human Subjects (IRB) 3535 Market Street, Suite 1200 Philadelphia, PA 19104 Tel: 215-590-2830 Email: [IRBOffice@emial.chop.edu](mailto:IRBOffice@emial.chop.edu) Email: [regaffairs@email.chop.edu](mailto:regaffairs@email.chop.edu) |
| **University of Pittsburgh** Institutional Review Board 3500 Fifth Avenue Pittsburgh, PA 15213 Tel: 412-383-1480 Fax: 412-383-1508 Web: <http://www.irb.pitt.edu> |
| **University of California** San Francisco Human Research Protection Program Committee on Human Research 3333 California St., Suite 315 San Francisco, CA 94118 Tel: 415-476-1814 Web: [IRB@ucsf.edu](mailto:IRB@ucsf.edu) |
| **Seattle Children’s** Hospital Research Foundation Institutional Review Board FWA: 00002443 P.O. Box 5371 Seattle, WA 98145 Tel: 206-987-7804 Web: [irb@seattlechildrens.org](mailto:irb@seattlechildrens.org) |
| Research Ethics Board **The Hospital for Sick Children** 555 University Avenue Toronto, Ontario Canada M5G 1X8 Tel: 416-813-8279 Fax: 416-813-6515 Web: [www.sickkids.ca](http://www.sickkids.ca) |
| **Washington University in St. Louis** Human Research Protection Office 660 South Euclid Ave., Campus Box 8089 St. Louis, MO 63110 Tel: 314-633-7400 Fax: 314-367-3041 Web: <http://hrpohome.wustl.edu/> |
| **University of Michigan** Medical School Institutional Review Board (IRBMED) 2800 Plymouth Road Building 520, Room 3214 Ann Arbor, MI 48109-2800 Tel: 734-763-4768 Fax: 734-763-9603 Web: [irbmed@umich.edu](mailto:irbmed@umich.edu) |
| Ann & Robert H. Lurie **Children’s Hospital of Chicago** Institutional Review Board 225 E Chicago Ave. Room #19-376 Chicago, IL 60611 Tel: 773-755-6301 Fax: 773-755-6533 Email: [IRB@Luriechildrens.org](mailto:IRB@Luriechildrens.org) |
| **The University of Utah** Institutional Review Board 75 South 2000 East Salt Lake City, UT 84112 Tel: 801-851-3655 Email: [IRB@utah.edu](mailto:IRB@utah.edu) |
