# Supplementary material for: Initial assessment of the infant with neonatal cholestasis—Is this biliary atresia?
Source: PLoS One. 2017 May 11;12(5):e0176275. doi: 10.1371/journal.pone.0176275 (PMC5426590; doi:10.1371/journal.pone.0176275)
Supplement: S2 Table — (DOCX) [file pone.0176275.s002.docx]

Supporting Information for:

**Initial assessment of the infant with neonatal cholestasis – is this biliary atresia?**

Benjamin L. Shneider^1*^, Jeff Moore^2^, Nanda Kerkar^3,4^, John C. Magee^5^, Wen Ye^2^, Saul J. Karpen^6^, Binita M. Kamath^7^, Jean P. Molleston^8^, Jorge A. Bezerra^9^, Karen F. Murray^10^, Kathleen M. Loomes^11^, Peter F. Whitington^12^, Philip Rosenthal^13^, Robert H. Squires^14^, Stephen L. Guthery^15^, Ronen Arnon^4^, Kathleen B. Schwarz^16^, Yumirle P. Turmelle^17^, Averell H. Sherker^18^, Ronald J. Sokol^19^ for the Childhood Liver Disease Research Network.

^1^ Pediatric Gastroenterology, Hepatology, and Nutrition; Baylor College of Medicine; Houston, Texas, United States

^2^ Department of Biostatistics; University of Michigan; Ann Arbor, Michigan, United States

^3^ Children’s Hospital of Los Angeles; Los Angeles, California, United States

^4^ Mount Sinai; New York, New York, United States

^5^ University of Michigan Medical School; Ann Arbor, Michigan, United States

^6^ Pediatric Gastroenterology, Hepatology, and Nutrition; Emory University School of Medicine/Children’s Healthcare of Atlanta; Atlanta, Georgia, United States

^7^ Division of Gastroenterology, Hepatology, and Nutrition; Hospital for Sick Children and University of Toronto; Toronto, Ontario, Canada

^8^ Pediatric Gastroenterology, Hepatology, and Nutrition; Indiana University School of Medicine/Riley Hospital for Children; Indianapolis, Indiana, United States

^9^ Division of Pediatric Gastroenterology, Hepatology, and Nutrition; Cincinnati Children’s Hospital Medical Center; Cincinnati, Ohio, United States

^10^ Division of Gastroenterology and Hepatology; University of Washington Medical Center, Seattle Children’s, Seattle, Washington, United States

^11^ Pediatric Gastroenterology, Hepatology, and Nutrition; Children’s Hospital of Philadelphia; Philadelphia, Pennsylvania, United States

^12^ Pediatrics Division of Gastroenterology, Hepatology, and Nutrtion; Ann and Robert H. Lurie Children’s Hospital of Chicago; Chicago, Illinois, United States

^13^ Division of Gastroenterology, Hepatology, and Nutrition; Department of Pediatrics; University of California, San Francisco; San Francisco, California, United States

^14^ Children’s Hospital of Pittsburgh; Pittsburgh, Pennsylvania, United States

^15^ Pediatric Gastroenterology, Hepatology, and Nutrition; University of Utah, Salt Lake City, Utah, United States

^16^ Johns Hopkins School of Medicine; Baltimore, Maryland, United States

^17^ Washington University School of Medicine, St. Louis, Missouri, United States

^18^ Liver Diseases Research Branch, National Institute of Diabetes and Digestive and Kidney Diseases, National Institutes of Health; Bethesda, Maryland, United States

^19^ Section of Pediatric Gastroenterology, Hepatology, and Nutrition, Department of Pediatrics, University of Colorado School of Medicine; Children’s Hospital Colorado; Aurora, Colorado, United States

***Corresponding Author:** Benjamin L. Shneider: [Benjamin.Shneider@bcm.edu](mailto:Benjamin.Shneider@bcm.edu)

**S2 Table. Comparison of included (Group 1) and excluded (Group 3) infants with a clinical diagnosis of biliary atresia**

| **Variable** | **Group 1 (BA Included) % or Mean (SD) N=401** | **Group 3 (BA Excluded) % or Mean (SD) N=102** | **p-value** |
| --- | --- | --- | --- |
| Race |  |  | 0.500 |
| White | 244 (63%) | 55 (57.3%) |  |
| Black | 61 (15.8%) | 19 (19.8%) |  |
| Asian | 36 (9.3%) | 7 (7.3%) |  |
| Other | 46 (11.9%) | 15 (15.6%) |  |
| Sex |  |  | 0.362 |
| Male | 191 (47.6%) | 43 (42.6%) |  |
| Female | 210 (52.4%) | 58 (57.4%) |  |
| Ethnicity |  |  | **0.009** |
| Hispanic | 92 (23%) | 36 (35.6%) |  |
| Non-Hispanic | 308 (77%) | 65 (64.4%) |  |
| Age at First Evaluation (Days) | N=401 | N=102 | **0.001** |
|  | 63.5 (30.9) | 79.7 (46.3) |  |
| Age at Disease Onset (Days) | N=401 | N=102 | **0.019** |
|  | 12.8 (18.5) | 19.9 (28.8) |  |
| Weight (kg) | N=398 | N=97 | **0.047** |
|  | 4.5 (0.9) | 4.8 (1.2) |  |
| Length (cm) | N=381 | N=91 | **0.039** |
|  | 55.5 (4) | 56.8 (5.5) |  |
| Head Circumference (cm) | N=336 | N=81 | 0.567 |
|  | 37.6 (2.2) | 37.8 (2.9) |  |
| Weight Z-Score | N=398 | N=96 | 0.152 |
|  | -1 (1) | -1.1 (1.1) |  |
| Length Z-Score | N=381 | N=90 | 0.610 |
|  | -0.8 (1.5) | -0.9 (1.6) |  |
| Head Circumference Z-Score | N=336 | N=81 | **0.066** |
|  | -1.1 (1.6) | -1.5 (1.7) |  |
| Acholic Stools |  |  | **0.043** |
| Absent | 69 (17.6%) | 25 (26.9%) |  |
| Present | 322 (82.4%) | 68 (73.1%) |  |
| Acholic Stools (3 Levels) |  |  | 0.127 |
| Normal | 69 (17.6%) | 25 (26.9%) |  |
| White or Gray | 184 (47.1%) | 38 (40.9%) |  |
| Pale | 138 (35.3%) | 30 (32.3%) |  |
| Facial Features |  |  | >0.999 |
| Normal | 380 (95.2%) | 94 (95.9%) |  |
| Abnormal | 19 (4.8%) | 4 (4.1%) |  |
| Liver Edge Palpable |  |  | **0.061** |
| Not Palpable | 26 (7.3%) | 10 (12%) |  |
| Palpable | 332 (92.7%) | 73 (88%) |  |
| Liver Edge Below Costal Margin (cm) | N=334 | N=73 | 0.112 |
|  | 3.3 (1.6) | 3.6 (1.7) |  |
| Spleen Palpable |  |  | **0.045** |
| Not Palpable | 188 (50%) | 34 (38.2%) |  |
| Palpable | 188 (50%) | 55 (61.8%) |  |
| Direct Baseline Bilirubin (mg/dL) | N=239 | N=36 | **0.015** |
|  | 5.7 (2.2) | 4.7 (3.1) |  |
| Conjugated Baseline Bilirubin (mg/dL) | N=215 | N=35 | **0.037** |
|  | 4.3 (1.6) | 3.8 (2) |  |
| Total Baseline Bilirubin (mg/dL) | N=401 | N=89 | 0.948 |
|  | 8.3 (3.1) | 8.8 (4.4) |  |
| AST (IU/L) | N=397 | N=91 | 0.261 |
|  | 232.1 (206.4) | 232.8 (209.5) |  |
| ALT (IU/L) | N=400 | N=92 | 0.157 |
|  | 154.7 (124.3) | 147.1 (129.7) |  |
| Albumin (g/dL) | N=391 | N=89 | 0.312 |
|  | 3.6 (0.5) | 3.8 (3.5) |  |
| GGTP (IU/L) | N=379 | N=78 | 0.218 |
|  | 711.9 (537.5) | 681.3 (600.9) |  |
| Platelets (10^3^/ mm^3^) | N=380 | N=88 | **0.008** |
|  | 445.2 (180.2) | 383.7 (153.4) |  |
| Alkaline Phosphatase (IU/L) | N=395 | N=87 | 0.789 |
|  | 568.6 (320.7) | 606 (421.5) |  |
| Total Cholesterol (mg/dL) | N=33 | N=13 | 0.657 |
|  | 184.2 (61.3) | 198.7 (76.2) |  |
| Gallbladder |  |  | 0.149 |
| Absent | 125 (39.9%) | 36 (55.4%) |  |
| Present | 5 (1.6%) | 0 (0.0%) |  |
| Present (Small) | 142 (45.4%) | 22 (33.8%) |  |
| Normal | 41 (13.1%) | 7 (10.8%) |  |
| Gallbladder (Absent vs. Present) |  |  | **0.022** |
| Absent | 125 (39.9%) | 36 (55.4%) |  |
| Present | 188 (60.1%) | 29 (44.6%) |  |
