# Supplementary material for: Initial assessment of the infant with neonatal cholestasis—Is this biliary atresia?
Source: PLoS One. 2017 May 11;12(5):e0176275. doi: 10.1371/journal.pone.0176275 (PMC5426590; doi:10.1371/journal.pone.0176275)
Supplement: S3 Table — (DOCX) [file pone.0176275.s003.docx]

Supporting Information for:

**Initial assessment of the infant with neonatal cholestasis – is this biliary atresia?**

Benjamin L. Shneider^1*^, Jeff Moore^2^, Nanda Kerkar^3,4^, John C. Magee^5^, Wen Ye^2^, Saul J. Karpen^6^, Binita M. Kamath^7^, Jean P. Molleston^8^, Jorge A. Bezerra^9^, Karen F. Murray^10^, Kathleen M. Loomes^11^, Peter F. Whitington^12^, Philip Rosenthal^13^, Robert H. Squires^14^, Stephen L. Guthery^15^, Ronen Arnon^4^, Kathleen B. Schwarz^16^, Yumirle P. Turmelle^17^, Averell H. Sherker^18^, Ronald J. Sokol^19^ for the Childhood Liver Disease Research Network.

^1^ Pediatric Gastroenterology, Hepatology, and Nutrition; Baylor College of Medicine; Houston, Texas, United States

^2^ Department of Biostatistics; University of Michigan; Ann Arbor, Michigan, United States

^3^ Children’s Hospital of Los Angeles; Los Angeles, California, United States

^4^ Mount Sinai; New York, New York, United States

^5^ University of Michigan Medical School; Ann Arbor, Michigan, United States

^6^ Pediatric Gastroenterology, Hepatology, and Nutrition; Emory University School of Medicine/Children’s Healthcare of Atlanta; Atlanta, Georgia, United States

^7^ Division of Gastroenterology, Hepatology, and Nutrition; Hospital for Sick Children and University of Toronto; Toronto, Ontario, Canada

^8^ Pediatric Gastroenterology, Hepatology, and Nutrition; Indiana University School of Medicine/Riley Hospital for Children; Indianapolis, Indiana, United States

^9^ Division of Pediatric Gastroenterology, Hepatology, and Nutrition; Cincinnati Children’s Hospital Medical Center; Cincinnati, Ohio, United States

^10^ Division of Gastroenterology and Hepatology; University of Washington Medical Center, Seattle Children’s, Seattle, Washington, United States

^11^ Pediatric Gastroenterology, Hepatology, and Nutrition; Children’s Hospital of Philadelphia; Philadelphia, Pennsylvania, United States

^12^ Pediatrics Division of Gastroenterology, Hepatology, and Nutrtion; Ann and Robert H. Lurie Children’s Hospital of Chicago; Chicago, Illinois, United States

^13^ Division of Gastroenterology, Hepatology, and Nutrition; Department of Pediatrics; University of California, San Francisco; San Francisco, California, United States

^14^ Children’s Hospital of Pittsburgh; Pittsburgh, Pennsylvania, United States

^15^ Pediatric Gastroenterology, Hepatology, and Nutrition; University of Utah, Salt Lake City, Utah, United States

^16^ Johns Hopkins School of Medicine; Baltimore, Maryland, United States

^17^ Washington University School of Medicine, St. Louis, Missouri, United States

^18^ Liver Diseases Research Branch, National Institute of Diabetes and Digestive and Kidney Diseases, National Institutes of Health; Bethesda, Maryland, United States

^19^ Section of Pediatric Gastroenterology, Hepatology, and Nutrition, Department of Pediatrics, University of Colorado School of Medicine; Children’s Hospital Colorado; Aurora, Colorado, United States

***Corresponding Author:** Benjamin L. Shneider: [Benjamin.Shneider@bcm.edu](mailto:Benjamin.Shneider@bcm.edu)

**S3 Table. Comparison of included (Group 2) and excluded (Group 4) infants with a clinical diagnosis that was not biliary atresia**

| **Variable** | **Group 2 (Non-BA Included) % or Mean (SD) N=259** | **Group 4 (Non-BA Excluded) % or Mean (SD) N=113** | **p-value** |
| --- | --- | --- | --- |
| Race |  |  | 0.280 |
| White | 156 (61.2%) | 60 (57.7%) |  |
| Black | 51 (20%) | 24 (23.1%) |  |
| Asian | 13 (5.1%) | 10 (9.6%) |  |
| Other | 35 (13.7%) | 10 (9.6%) |  |
| Sex |  |  | 0.880 |
| Male | 164 (63.3%) | 70 (62.5%) |  |
| Female | 95 (36.7%) | 42 (37.5%) |  |
| Ethnicity |  |  | 0.309 |
| Hispanic | 60 (23.3%) | 20 (18.5%) |  |
| Non-Hispanic | 197 (76.7%) | 88 (81.5%) |  |
| Age at First Evaluation (Days) | N=259 | N=113 | 0.332 |
|  | 60 (33.3) | 56.4 (33) |  |
| Age at Disease Onset (Days) | N=259 | N=113 | 0.371 |
|  | 18.7 (22.1) | 21 (25.1) |  |
| Weight (kg) | N=257 | N=102 | 0.436 |
|  | 4.1 (1.1) | 4.2 (1.1) |  |
| Length (cm) | N=252 | N=99 | 0.970 |
|  | 54.3 (4.3) | 54.3 (5.3) |  |
| Head Circumference (cm) | N=215 | N=81 | 0.688 |
|  | 37.1 (2.6) | 37.3 (2.3) |  |
| Weight Z-Score | N=257 | N=102 | 0.181 |
|  | -1.5 (1.2) | -1.3 (1.3) |  |
| Length Z-Score | N=252 | N=99 | 0.928 |
|  | -1.4 (1.5) | -1.3 (2.1) |  |
| Head Circumference Z-Score | N=215 | N=81 | 0.170 |
|  | -1.4 (1.2) | -1.2 (1.3) |  |
| Acholic Stools |  |  | 0.254 |
| Absent | 165 (66%) | 73 (72.3%) |  |
| Present | 85 (34%) | 28 (27.7%) |  |
| Acholic Stools (3 Levels) |  |  | 0.335 |
| Normal | 165 (66%) | 73 (72.3%) |  |
| White or Gray | 30 (12%) | 7 (6.9%) |  |
| Pale | 55 (22%) | 21 (20.8%) |  |
| Facial Features |  |  | **0.070** |
| Normal | 207 (81.2%) | 90 (89.1%) |  |
| Abnormal | 48 (18.8%) | 11 (10.9%) |  |
| Liver Edge Palpable |  |  | **0.001** |
| Not Palpable | 34 (14.5%) | 28 (30.1%) |  |
| Palpable | 201 (85.5%) | 65 (69.9%) |  |
| Liver Edge Below Costal Margin (cm) | N=202 | N=65 | 0.513 |
|  | 2.5 (1.4) | 2.4 (1.3) |  |
| Spleen Palpable |  |  | 0.326 |
| Not Palpable | 149 (59.6%) | 64 (65.3%) |  |
| Palpable | 101 (40.4%) | 34 (34.7%) |  |
| Direct Baseline Bilirubin (mg/dL) | N=162 | N=51 | **<0.001** |
|  | 5.8 (3.2) | 3.8 (3.2) |  |
| Conjugated Baseline Bilirubin (mg/dL) | N=121 | N=38 | **0.014** |
|  | 4.6 (2.6) | 3.4 (2.2) |  |
| Total Baseline Bilirubin (mg/dL) | N=259 | N=95 | **0.002** |
|  | 8.6 (4.3) | 7.6 (5.1) |  |
| AST (IU/L) | N=254 | N=92 | **<0.001** |
|  | 284.2 (347.7) | 189.6 (189.7) |  |
| ALT (IU/L) | N=255 | N=97 | **0.001** |
|  | 190.7 (232.5) | 137.2 (179.5) |  |
| Albumin (g/dL) | N=246 | N=94 | **0.040** |
|  | 3.5 (0.6) | 4.1 (5) |  |
| GGTP (IU/L) | N=238 | N=88 | 0.126 |
|  | 299 (380.5) | 327.8 (356.3) |  |
| Platelets (10^3^/ mm^3^) | N=243 | N=87 | 0.666 |
|  | 419.7 (197.3) | 422.1 (181.7) |  |
| Alkaline Phosphatase (IU/L) | N=254 | N=94 | **0.056** |
|  | 572.1 (252.1) | 522.9 (257.2) |  |
| Total Cholesterol (mg/dL) | N=54 | N=15 | 0.725 |
|  | 190.6 (82.3) | 185.3 (87) |  |
| Gallbladder |  |  | 0.213 |
| Absent | 13 (6.5%) | 8 (11.1%) |  |
| Present | 1 (1.4%) | 0 (0.0%) |  |
| Present (Small) | 81 (40.7%) | 29 (40.3%) |  |
| Normal | 105 (52.8%) | 34 (47.2%) |  |
| Gallbladder (Absent vs. Present) |  |  | 0.209 |
| Absent | 13 (6.5%) | 8 (11.1%) |  |
| Present | 186 (93.5%) | 64 (88.9%) |  |
